# Wound infection with *Vibrio harveyi* following a traumatic leg amputation after a motorboat propeller injury in Mallorca, Spain: a case report and review of literature

**DOI:** 10.1186/s12879-020-4789-2

**Published:** 2020-02-04

**Authors:** Thomas Theo Brehm, Laura Berneking, Holger Rohde, Martin Chistner, Carsten Schlickewei, Meike Sena Martins, Stefan Schmiedel

**Affiliations:** 1 Division of Infectious Diseases, I. Department of Internal Medicine, University Medical Center Hamburg-Eppendorf, Hamburg, Germany; 2 German Center for Infection Research (DZIF), Partner Site Hamburg-Lübeck-Borstel-Riems, Hamburg, Germany; 3 Institute of Medical Microbiology, Virology and Hygiene, University Medical Center Hamburg-Eppendorf, Hamburg, Germany; 4 Department of Trauma, Hand, and Reconstructive Surgery, University Medical Center Hamburg-Eppendorf, Hamburg, Germany; 5 Institute of Oceanography, University of Hamburg, Hamburg, Germany

**Keywords:** *Vibrio harveyi*, *Vibrio carchariae*, Non-cholera *Vibrio*, Amputation injury, Motorboat propeller injury, Wound infection, Mediterranean Sea, Climate change, Global warming, Sea surface temperature

## Abstract

**Background:**

*Vibrio* spp. are aquatic bacteria that are ubiquitous in warm estuarine and marine environments, of which 12 species are currently known to cause infections in humans. So far, only five human infections with *V. harveyi* have been reported.

**Case presentation:**

A 26-year old patient was transferred to our center by inter-hospital air transfer from Mallorca, Spain. Seven days before, he had suffered a complete amputation injury of his left lower leg combined with an open, multi-fragment, distal femur fracture after he had been struck by the propeller of a passing motorboat while snorkeling in the Mediterranean Sea. On admission he was febrile; laboratory studies showed markedly elevated inflammatory parameters and antibiotic treatment with ampicillin/sulbactam was initiated. Physical examination showed a tender and erythematous amputation stump, so surgical revision was performed and confirmed a putrid infection with necrosis of the subcutaneous tissue and the muscles. Tissue cultures subsequently grew *V. harveyi* with a minimal inhibitory concentration (MIC) of 16 mg/L for ampicillin, and antibiotic treatment was switched to ceftriaxone and ciprofloxacin. Throughout the following days, the patient repeatedly had to undergo surgical debridement but eventually the infection could be controlled, and he was discharged.

**Conclusions:**

We report the first human infection with *V. harveyi* acquired in Spain and the second infection acquired in the Mediterranean Sea. This case suggests that physicians and microbiologists should be aware of the possibility of wound infections caused by *Vibrio* spp. acquired in the ocean environment, especially during hot summer months. Since *Vibrio* spp. preferentially grow at water temperatures above 18 °C, global warming is responsible for an abundance of these bacteria in coastal waters. This will likely lead to a worldwide increase in reports of *Vibrio*-associated diseases in the future.

## Background

*Vibrio* is a genus of Gram-negative, rod-shaped bacteria that can be found in a wide variety of estuarine and marine environments. Of the > 100 described *Vibrio* spp., at least 12 species are currently known to have the potential to cause human infections (Table 1).

**Table 1:** *Vibrio spp.* that cause, or are associated with, human infections (modified from [1])

Species	Occurrence in clinical specimens	
	Intestinal	Extraintestinal
*V. alginolyticus*	–	++
*V. cholerae*		
O1/O139	++++	+
non-O1/O139	++	++
*V. cincinnatiensis*	–	+
*V. damsela*	–	+
*V. fluvialis*	++	–
*V. furnissii*	++	–
*V. harveyi*	–	+
*V. hollisae*	++	–
*V. metschnikovii*	+	+
*V. mimicus*	++	+
*V. parahaemolyticus*	++++	+
*V. vulnificus*	+	+++

The symbols +, ++, +++ and ++++ give the relative frequency of each organism in the specimens; −, not found

The toxin-producing serogroups O1 and O139 of *V. cholerae* can cause endemic and epidemic cholera, a severe acute secretory diarrheal illness that is primarily transmitted via ingestion of contaminated food or water. Non-cholera *Vibrio* spp. infections are most often caused by halophilic species that thrive in saltwater. Thus, these infections are usually associated with consumption of raw or undercooked seafood or traumatic exposure to sea or brackish water and can to a variable degree cause gastroenteritis and wound infections [1–3]. *V. harveyi* (synonym *V. carchariae*) is known to be one of the causative agents of systemic fish disease as well as seafood spoilage [4, 5] and has long been considered non-pathogenic to humans [6]. However, sporadic case reports about human infections have been published in the recent years. The purpose of this study was to report the second human infection with *V. harveyi* acquired in the Mediterranean Sea in order to increase alertness in physicians and microbiologists, since the ongoing global warming will likely lead to a worldwide increase in *Vibrio*-associated infections.

## Case presentation

**Fig. 1:**
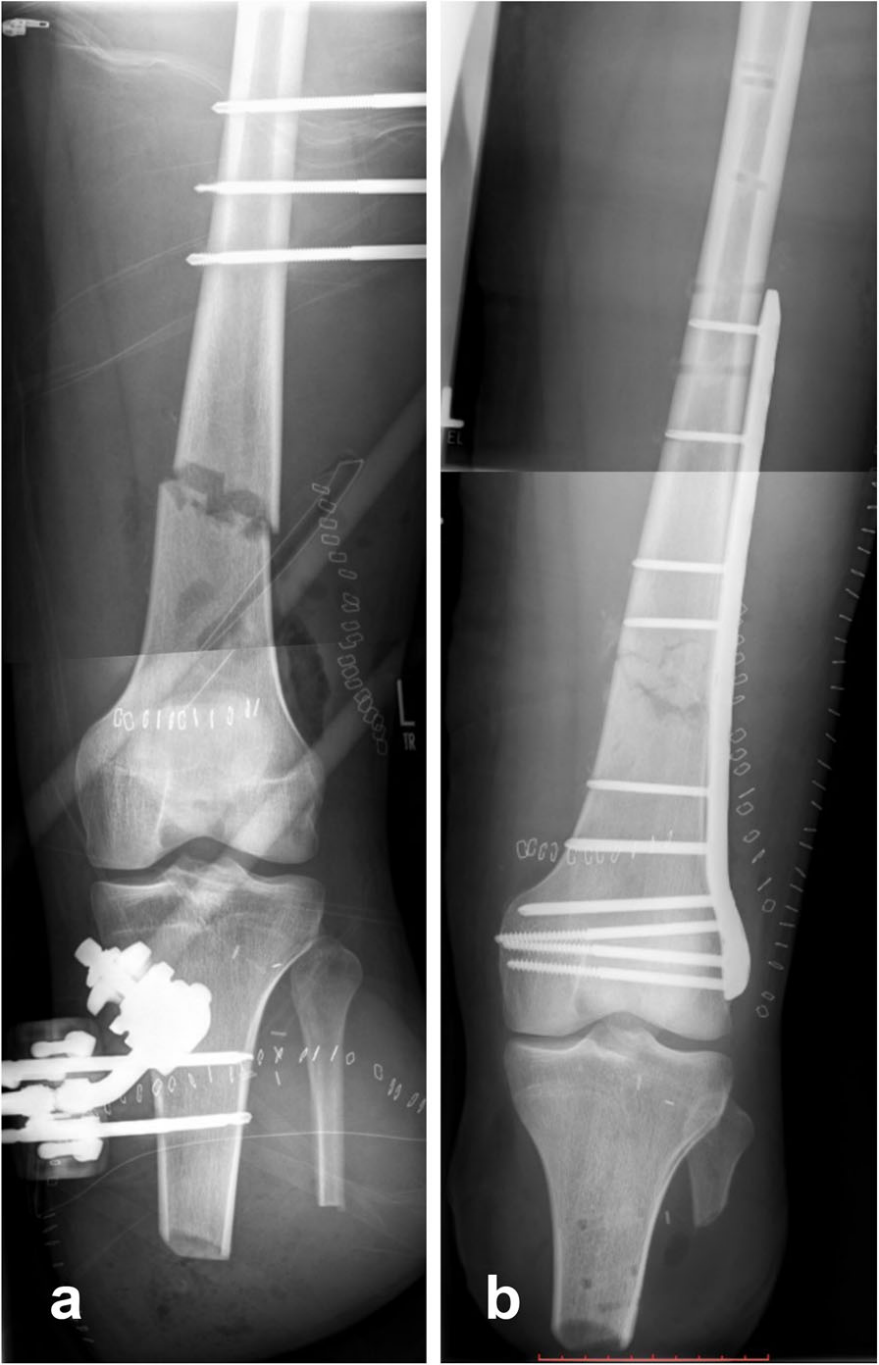
Anterior posterior X-ray of the left lower extremity. **a** After transport of the patient from Spain to our hospital, with lower leg stump and external fixator, after traumatic amputation of the lower leg and open, multi-fragmentary, distal femoral fracture (day 1). **b** After definitive surgery, with a closed, infection-free amputation stump and after open reduction and stabilization of the multi-fragment distal femur fracture with an angular stable plate osteosynthesis (day 20)

During July 2018, a 26-year old, previously healthy male had been struck by the propeller of a passing motorboat while snorkeling on the shore of the Balearic island of Mallorca, Spain. He suffered complete amputation injury at the level of the proximal tibia and fibula as well as multi-fragment distal femur fracture of his left lower extremity (Fig. 1). Due to retrograde amnesia the patient was not able to provide more detailed information concerning the accident occurrence. At the local hospital, the lower leg showed extensive soft-tissue damage and had to be amputated. The open, multi-fragment distal femur fracture was reduced and stabilized with an external fixator. Postoperatively, the patient was transferred to an intensive care unit. Seven days after the injury, on August 02, 2018 the patient was presented to our center by inter-hospital air transfer. On admission he was febrile with a temperature of 38.5 °C. Laboratory studies revealed a normal white blood cell count of 7.3 × 10^9^/l (reference 3.8–11.0 × 10^9^/l), anemia with hemoglobin level of 10.0 g/dl (reference 14.0–17.5 g/dl) and elevated C-reactive protein of 253 mg/l (reference < 5 mg/l). Chest radiograph and urine analysis showed no abnormalities and four sets of blood culture showed no growth of bacteria. Initially the amputation stump and the wound on the thigh showed no clinical signs of infection. Blood cultures were collected before empiric antimicrobial therapy was initiated with ampicillin/sulbactam. Two days later clinical examination revealed a warm, tender and erythematous amputation stump; hence a wound infection was suspected. On August 04, 2018 surgical revision revealed a putrid infection of the amputation stump with necrosis of the subcutaneous tissue and the muscles. Due to the advanced infection, a rigorous and aggressive debridement of skin, subcutaneous tissue, fascia, muscle and bone was performed. The amputation stump was temporarily closed with a vacuum assisted closure-therapy. The external fixator was removed, and the pin sites and the wound were cut out. Subsequently, the multi-fragmented distal femur fracture was reduced and stabilized with a new external fixator. During the surgery several microbiological samples were submitted for microbiologic examination. Culture incubation of a wound swab readily grew bacteria that were identified as *V. harveyi* by matrix-assisted laser desorption ionization/time-of-flight mass spectrometry fingerprinting using a biotyper instrument (Bruker Daltonics, Bremen, Germany). The wound culture was monomicrobial without growth of any other pathogens. Minimal inhibitory concentrations (MICs) were determined by gradient diffusion (Etest®, bioMérieux, Marcy-l’Étoile, France; MIC Test Strip, Liofilchem, Roseto degli Abruzzi, Italy). According to non-species related EUCAST breakpoints, the isolate was classified as susceptible to meropenem (MIC 0.004 mg/mL), ciprofloxacin (0.125 mg/mL) and ceftriaxone (0.032 mg/mL) and resistant to ampicillin (32 mg/mL) (Table 2).

**Table 2:** Antimicrobial susceptibility of *Vibrio harveyi* with interpretation according to non-species related EUCAST breakpoints

Antimicrobial agent	MIC (μg/ml)	Interpretation
Amikacin	8	
Amoxicillin	16	R
Ampicillin	16	R
Azithromycin	2	
Aztreonam	2	S
Ciprofloxacin	0.125	S
Ceftriaxon	0.125	S
Erythromycin	4	
Gentamicin	1	
Imipenem	0.125	S
Meropenem	0.064	S
Moxifloxacin	0.25	
Penicillin	> 32	R
Piperacillin/Tazobactam	0.125	S
TMP/SMX	0.032	
Doxycycline	0.25	
Tobramycin	2	
Tetracyclin	0.25	
Streptomycin	16	

*I* intermediate, *MIC* minimum inhibitory concentration, *R* resistant, *S* susceptible, *TMP/SMX* trimethoprim/sulfamethoxazole

Thus, antibiotic treatment was transitioned to ceftriaxone and ciprofloxacin. Throughout the following days, the patient repeatedly had to undergo surgical debridement, but the infection could eventually be controlled. Sixteen days after the first surgical revision the amputation stump could be closed, and the multi-fragment distal femur fracture was stabilized with a 9-hole NCB distal femur plate (Fig. 1b). On September 04, 2018, antibiotic treatment was stopped, and the patient was discharged to a rehabilitation facility.

Whole genome sequencing of the isolate was retrospectively performed with an Illumina NextSeq instrument using 2 × 150 bp paired-end chemistry (SRA accession PRJNA576306). Reads were assembled with shovill 1.0.4 (https://github.com/tseemann/shovill) and spades 3.13.1 [7] into a draft genome with 107 contigs larger than 200 bp (N50 409272, L50 4) and a total length of 6.1 million bp. An average nucleotide identity of 98.6 to 98.8 with genomes of *V. harveyi* strains 345, ATCC 33843, CAIM 1792, NCTC 12970, VHJR4 and QT520 confirmed the species identification [8]. RAST-annotation [9] and analysis with the SEED-viewer [10] showed a nearly identical profile of the investigated *V. harveyi* strains in the virulence, disease and defense subsystem category. An additional blast-search against the virulence factor database [11] did not reveal additional atypical virulence factors (e. g. toxins) in our isolate. In agreement with phenotypic testing (Table 2) resistance gene profiling identified a class A beta-lactamase showing 100% amino acid identity to the beta-lactamase of *V. harveyi* CAIM 1792 (GenBank® accession number EMR34292.1).

## Discussion and conclusion

So far, only five human infections caused by *V. harveyi* have been reported. The first report described a wound infection acquired by an 11-year-old girl after being attacked by a shark while wading in knee-deep water off the South Carolina coast [12]. The second case was an episode of catheter-related bacteremia in a 9-year old British oncologic patient with a central line after returning from a holiday in Perpignan, France, where he had swum in the Mediterranean Sea [13]. Both a 64-year-old and a 75-year-old man acquired mixed wound infections with *Photobacterium damselae* and *V. harveyi* in Australia after experiencing a laceration injury after falling from a catamaran and after fishing without recalling any trauma respectively [14, 15]. Another wound infection with *V. harveyi* occurred in a 49-year old immunosuppressed Spanish traveler in the Dominican Republic after experiencing trauma from striking a bus step and subsequently swimming in the sea [16].

In our patient, culture incubation of a wound swab grew *V. harveyi* on August 04, 7 days after the initial injury and admission to the local hospital. The culture was monomicrobial without growth of any other pathogens. It is beyond our knowledge whether tissue cultures were performed prior to transferal to our hospital. However, a wound contamination is highly unlikely given that *V. harveyi* is a halophilic bacterium that only grows in salt- or brackish water.

**Fig. 2:**
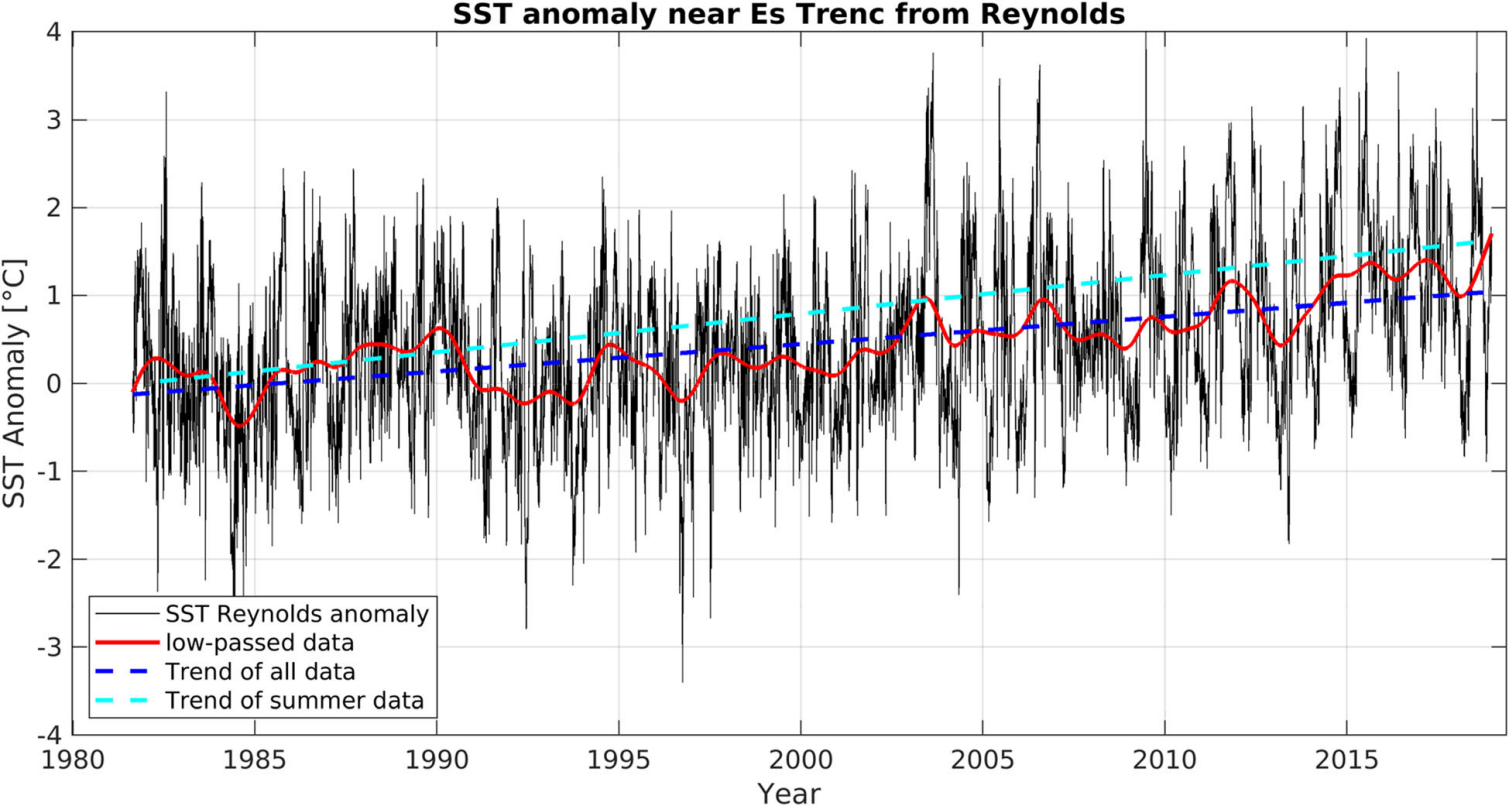
SST anomaly off the southern coast of Mallorca 1982–2018. SST anomaly (black) off the southern coast of Mallorca, with the low-passed data (> 1.6 years, red), the linear warming trend of all data (blue broken line), and the warming trend in the summer months (June, July and August, light blue line). For Methods see Additional file 1

**Fig. 3:**
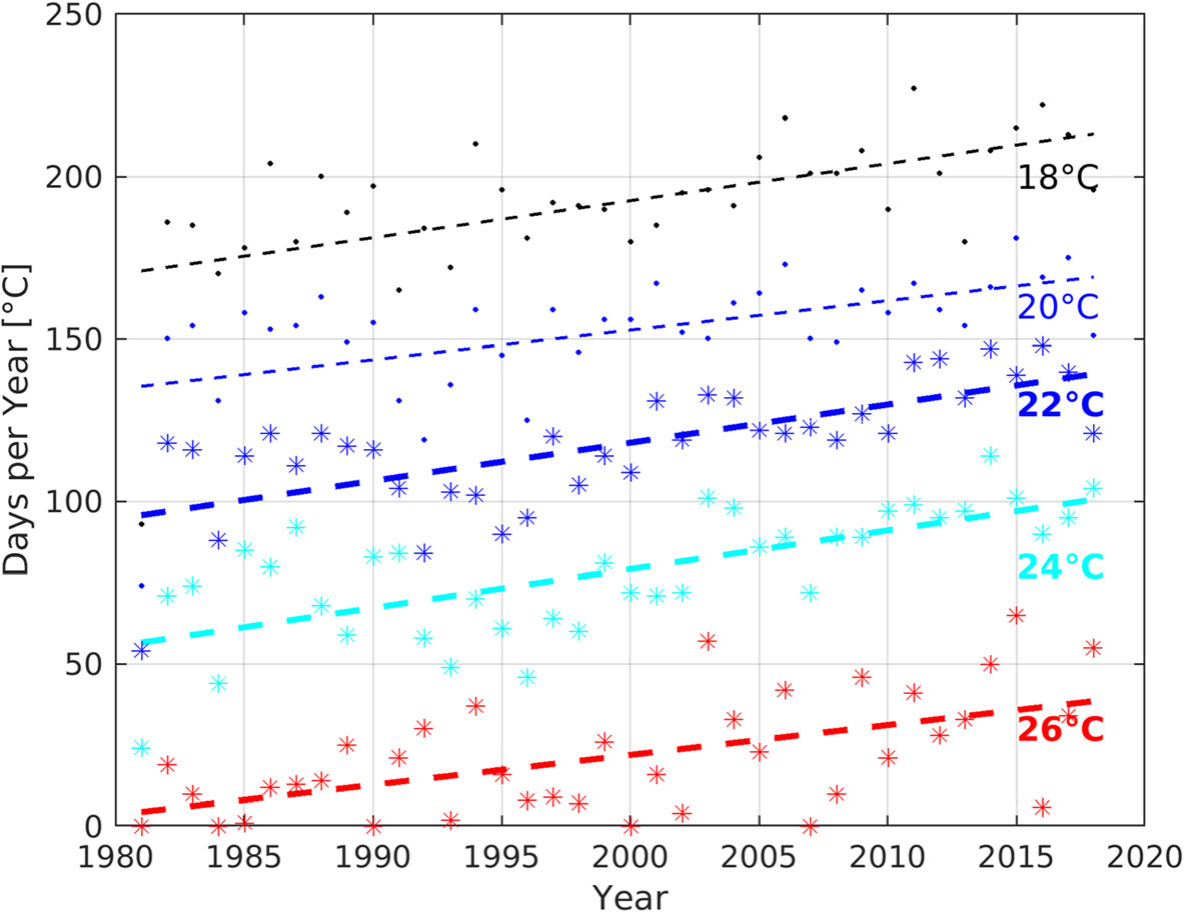
Absolute SST off the southern coast of Mallorca 1982–2018. Number of days with Reynolds SST off the southern coast of Mallorca exceeding 18 °C (black), 20 °C (blue, thin), 22 °C (blue), 24 °C (cyan) and 26 °C (red). The symbols denote the number of days per year, whereas the broken line shows the respective trend from 1982 to end 2018. For Methods see Additional file 1

Not all strains of *V. harveyi* are virulent. However, despite the role of the bacteria as a pathogen for both a wide range of both marine animals and humans, the pathogenicity mechanisms have not been fully understood yet [17]. The range of virulence determinants include among others the ability to attach and form biofilms, lipopolysaccharide, proteases, hemolysins, bacteriophage interaction and quorum sensing. Virulence factors of other *Vibrio* spp. like *V. vulnificus* also include an anti-phagocytic polysaccharide capsule, a variety of extracellular toxins and iron utilization [18]. All *Vibrio* spp. preferentially grow at water temperatures above 18 °C and for *V. harveyi* the optimal temperature for reproduction is 26 °C [19, 20]. Therefore, most cases of *Vibrio* infections have usually been reported in tropical or subtropical regions [21]. However, climate change is responsible for a rapidly warming marine environment globally and especially in European seas [22]. During the last decades the warming trend of the Mediterranean reached 0.36 °C per decade, exceeding the global ocean average of 0.15 °C per decade [23, 24]. The mean sea surface temperature (SST) off the south coast of the Balearic island of Mallorca, Spain during the summer months (June, July and August) has even increased by 0.51 °C per decade since 1980 (Fig. 2, Additional file 1). During the 1980s the SST in the Mediterranean amounted more than 26 °C only on 8 days per year on average. In contrast, during the recent 8 years an SST of more than 26 °C was measured on an average of 35 days per year (Fig. 3). Thus, the number of days on which the SST is suitable for growth of *Vibrio* spp. in general and *V. harveyi* in particular (SST > 26 °C) has steadily increased. The warming trend for the Mediterranean Sea will not decelerate for the next 100 years and the SST projected by these climate studies for the end of the current century is controlled mainly by emission variations rather than seasonal or regional variations [25]. This is likely to lead to a further increase in the abundance of those bacteria in the surface water and subsequently to an increase of human infections. However, while cholera infection due to *V. cholerae* (O1, O139) is a reportable disease in all European countries, infections with other *Vibrio* spp. are not reportable in all countries. This lack of mandatory notification systems prevents accurate estimates for infections with *Vibrio* spp. in Europe and a high number of unreported infections must be assumed [26]. In the Baltic Sea, where the warming trend is even 0.6 °C per decade, this increase in SST already has been linked to an increase of domestically acquired human infections with *Vibrio* spp. in several littoral states, most likely also since national quality of monitoring in littoral states is higher than in Mediterranean countries [27–29]. Outbreaks with high numbers of reported cases have been reported during heat waves, when recreational exposure to water, which appears to be responsible for a sizeable proportion of reported infections, also substantially increases [27].

Another factor that has been identified to play a role in controlling abundance of *Vibrio* spp. is sea salinity. Each *Vibrio* spp. is associated with a distinct, species-specific salinity range [30]. While *V. parahaemolyticus* (5–30 psu [practical salinity units]) [31], *V. vulnificus* (8–16 psu) [32] and *V. cholerae* (0–20 psu) [30] most commonly thrive at relatively low salinity, *V. harveyi* preferentially grows at relatively higher salinities of 30 to 40 psu [33]. The Mediterranean Sea has a salinity of 38 psu, which is the highest of all European seas and represents optimal growth conditions for *V. harveyi.*

While we report only the second human infection with *V. harveyi* acquired in the Mediterranean Sea, this species has been reported to be responsible for mass mortality events in benthic invertebrates in the Mediterranean Sea and to remain a major threat to the survival of those animals due to potential climate change related disease outbreaks [34].

In light of the high fatality rates associated with wound infections caused by *Vibrio* spp., early and aggressive antimicrobial therapy is essential. Clinical data from retrospective studies on patients with wound infections or septicemia by *V. vulnificus* support a combination antibiotic therapy with a third-generation cephalosporin plus a tetracycline or fluoroquinolone. This regimen can be employed as initial calculated therapy if an infection with *Vibrio* spp. is suspected. However, during the last decades antimicrobial resistance against all available antibiotics including carbapenems has been shown in different *Vibrio* spp. and multidrug resistant strains have become a significant healthcare concern [35–38].

In conclusion, physicians and microbiologists should be aware that *Vibrio* spp. are emerging pathogens and due to ongoing global warming, will likely lead to a worldwide increase in reports of *Vibrio*-associated wound infections and gastrointestinal infections acquired also in European seas including the Mediterranean.

## Supplementary information

**Additional file 1.** Methods for Figure 2 and Figure 3.

## Data Availability

Data sharing is not applicable to this article as no datasets were generated or analyzed during the current study.

## Abbreviations

MIC: Minimal inhibitory concentration
psu: Practical salinity units
SST: Sea surface temperature
