# Supplementary material for: Wound infection with Vibrio harveyi following a traumatic leg amputation after a motorboat propeller injury in Mallorca, Spain: a case report and review of literature
Source: BMC Infect Dis. 2020 Feb 4;20:104. doi: 10.1186/s12879-020-4789-2 (PMC7001194; doi:10.1186/s12879-020-4789-2)
Supplement: Supplementary file 1 — Additional file 1. Methods for Figure 2 and Figure 3. [file 12879_2020_4789_MOESM1_ESM.docx]

Additional file 1

The sea surface temperature (SST) at the place where the accident happened (39.3 °N, 2.7 °E) was extracted from a data set comprising satellite data (AVHRR) products that have been developed using optimum interpolation. The daily data span the period from September 1981 to the end of 2018. Linear regressions were performed for the SST anomaly, i.e., the mean annual cycle was subtracted from the 38-year time series, and for the number of days per year with SST exceeding 18 °C to 26 °C. The air temperature from a reanalysis product from the National Centers for Atmospheric Prediction (NCEP) and the National Center for Atmospheric Research (NCAR) was used for the long-term time temperature series off the southern coast of Mallorca.
